# Prophylactic Aspirin Use Following Shoulder Surgery: A Retrospective Cohort Study

**DOI:** 10.7759/cureus.115686

**Published:** 2026-09-02

**Authors:** Kyle A Scarano, Bradley Brickman, Ethan Sawyer, John Duldner, Elizabeth A Halstead, Patrick T Gorman, Vithal Shendge, Osama Elattar, David Sohn, Jacob D Miller

**Affiliations:** 1 Orthopaedic Surgery, Brown University, Providence, USA; 2 Department of Orthopaedic Surgery, University of Toledo Medical Center, Toledo, USA; 3 Sports Medicine, Twin Cities Orthopedics, Minneapolis, USA

**Keywords:** aspirin, dvt, hematoma, prophylaxis, shoulder arthroplasty, shoulder arthroscopy, vte

## Abstract

Background

There is no clear consensus regarding the role of aspirin (acetylsalicylic acid, ASA) for venous thromboembolism (VTE) prophylaxis following shoulder arthroscopy or arthroplasty. Although rare, catastrophic complications have been reported surrounding the use of aspirin, or lack thereof, including deep vein thrombosis (DVT), hematoma, hospital readmission, and reoperation.

Methodology

We conducted a retrospective review of 1,272 patients who underwent shoulder arthroscopy or arthroplasty at a single institution between 2017 and 2023. Baseline demographic information, medical risk factors, chronic anticoagulation status, and the use of postoperative VTE/DVT prophylaxis were collected. Outcomes recorded within 90 days included VTE/DVT, symptomatic hematoma, readmission, and reoperation. Patients were stratified by procedure type and ASA use.

Results

Postoperatively, 70.8% of patients received ASA, which was not significantly associated with DVT (0.24% overall incidence, p = 0.9864), hematoma (p = 0.7254), reoperation (p = 0.2901), or readmission (p = 0.4326). Compared with arthroscopy, arthroplasty was associated with significantly higher rates of hematoma (2.46% vs. 0.19%, p < 0.0001), reoperation (4.43% vs. 1.03%, p = 0.0004), and readmission (5.91% vs. 0.37%, p < 0.0001). Chronic anticoagulation independently increased the risk of readmission (p = 0.0014) and reoperation (p = 0.0176), while advanced age was associated with higher rates of hematoma (p = 0.0027) and readmission (p = 0.0077). No significant associations were found between body mass index or diabetes and adverse outcomes.

Conclusions

The observed incidence of symptomatic VTE/DVT following shoulder arthroscopy and arthroplasty was low. Postoperative ASA use was not significantly associated with VTE/DVT or the evaluated postoperative complications. However, given the limited number of VTE/DVT events and nonrandomized treatment allocation, these findings do not establish equivalence between prophylaxis strategies or exclude a clinically meaningful benefit of ASA. Decisions regarding chemoprophylaxis should therefore be individualized according to patient and procedural risk factors.

## Introduction

Venous thromboembolism/deep vein thrombosis (VTE/DVT) after surgery represents a significant source of postoperative morbidity and healthcare expenditure [1]. While VTE/DVT risk is well characterized in lower extremity surgery, risk factors and prevention strategies for shoulder surgery remain less clearly defined [1,2]. The incidence of symptomatic VTE/DVT following shoulder arthroplasty and arthroscopy has been reported as low as 0.24-2.6% and 0.01-0.25%, respectively [1]. Despite these relatively low rates, prophylactic strategies remain variable, and consensus regarding routine pharmacologic thromboprophylaxis has not been well established.

In at least two instances, changes in national guidelines for VTE/DVT prevention after shoulder surgery have been shown to correlate with its reduction as well as the reduction of mortality. Agarwal et al. showed that the rate and burden of VTE/DVT following shoulder arthroplasty decreased in the United States following the American Academy of Orthopaedic Surgeons’ (AAOS) adoption of new guidelines for prophylaxis in 2009. Aspirin (acetylsalicylic acid, ASA) usage also increased considerably over the same time period [3]. Similarly, Jameson et al. examined VTE/DVT rates after shoulder surgery in England and Wales after the National Institute for Health and Clinical Excellence (NICE) recommended using low-molecular-weight heparin (LMWH) for orthopedic inpatients in 2007. They found a significant decrease in mortality after shoulder replacement following the implementation of the guidelines [4].

However, there remains a risk with ASA as chemoprophylaxis for shoulder surgery. Anticoagulation medications after shoulder surgery have been shown to increase the risk of complications, including infection and revision rates [5]. One study demonstrated a significant increase in wound complications, infection rates, and the need for revision surgery when several anticoagulation therapies were utilized [6]. Despite the possible risks associated with ASA use, a retrospective review by Kirsch et al. demonstrated low rates of VTE/DVT when low-dose aspirin was given after shoulder arthroplasty as well as low rates of complications associated with the medication [7]. Additionally, multiple studies and reviews of VTE/DVT-related complications in shoulder surgery have failed to show that pharmacological prophylaxis provides a significant difference in rates of VTE/DVT following shoulder arthroscopy or arthroplasty [8-12]. As such, further study of the role of ASA in the prevention of these complications following shoulder surgery is necessary [13]. We hypothesized that postoperative aspirin use would not be associated with a detectable reduction in symptomatic VTE/DVT or an increase in bleeding-related complications. The primary objective of this study was to evaluate the association between postoperative aspirin use and symptomatic VTE/DVT, hematoma, readmission, and reoperation following shoulder arthroscopy and arthroplasty. A secondary objective was to evaluate demographic and clinical factors associated with these postoperative outcomes. Comparison of individual aspirin dosing or duration regimens was not a prespecified objective.

## Materials and methods

A retrospective cohort study was performed to assess potential risks and benefits associated with aspirin use within 90 days of shoulder arthroscopy or arthroplasty. The four primary outcomes examined were VTE/DVT, hematoma formation, 90-day readmission, and 90-day reoperation. All patients who underwent shoulder arthroplasty and/or shoulder arthroscopy between 2017 and 2023 at our academic medical center were included. Patients were excluded if they were unable to be followed for at least 90 days following the procedure and/or if insufficient outcome data were available from the electronic medical record. A total of 1,272 patients met the inclusion criteria. There were 203 arthroplasties and 1,069 arthroscopies. A standardized data collection tool was utilized. All data points were collected directly from the patient’s electronic medical records. Eligible patients were identified using CPT codes. Patients were excluded if 90-day follow-up or sufficient outcome documentation was unavailable. Included procedures were identified using Current Procedural Terminology (CPT) codes and confirmed by review of the operative report. The arthroscopy cohort included rotator cuff repair, labral repair and stabilization, debridement, subacromial decompression, distal clavicle excision, biceps procedures, lysis of adhesions, and other arthroscopic or arthroscopy-associated shoulder procedures, as detailed in Table 1.

**Table 1:** Shoulder procedures and corresponding Current Procedural Terminology (CPT) codes included in the study.

Procedure category	CPT code
Rotator cuff repair	29827
Labral repair	29806, 29807
Shoulder debridement	29822, 29823
Synovectomy	29820, 29821
Subacromial decompression	29826
Distal clavicle excision	29824
Biceps tenodesis	29828, 23430
Loose/Foreign body removal	29819
Lysis of adhesions	29825
Capsulorrhaphy	29806
Other arthroscopic	29805, 29999
Total shoulder arthroplasty	23472
Reverse total shoulder arthroplasty	23472

Open procedures performed concomitantly with or as part of an arthroscopy-based treatment episode, including open biceps tenodesis, were retained within the arthroscopy cohort. The arthroplasty cohort included both anatomic and reverse total shoulder arthroplasty (CPT 23472). Patients were further stratified according to whether postoperative aspirin prophylaxis was administered. Aspirin administration was nonrandomized and determined by individual surgeon practice and clinical judgment. ASA dosing was surgeon-dependent and consisted of either 81 mg twice daily or 325 mg daily for two to four weeks postoperatively. The study was not designed or powered to compare individual ASA doses or treatment durations.

Primary clinical variables collected included age, body mass index (BMI), gender, smoking history, presence of diabetes, and chronic anticoagulant use. Existing use of aspirin or anticoagulants was assessed. Chronic anticoagulation included vitamin K antagonists, direct oral anticoagulants, heparin-based anticoagulants, and non-aspirin antiplatelet agents; aspirin use was recorded separately. Perioperative interruption and resumption of chronic anticoagulation followed standard guideline-recommended intervals according to medication class and procedural bleeding risk. Use of mechanical prophylaxis and postoperative mobilization protocols could not be reliably determined retrospectively and therefore were not included as study variables. VTE/DVT events were identified based on symptomatic clinical presentation followed by confirmatory diagnostic imaging; routine screening of asymptomatic patients was not performed. DVT was confirmed by venous duplex ultrasonography, while pulmonary embolism was confirmed by computed tomography pulmonary angiography or other documented diagnostic imaging. Postoperative hematoma was defined as a clinically documented surgical-site hematoma identified during the 90-day postoperative period. Study outcomes occurring within 90 days of the primary procedure were collected from office visits (specialty clinic or primary care), emergency department visits, hospital admissions, or subsequent operating room documentation.

Statistical analysis

All statistical analyses were performed in consultation with a statistician from our institution using SAS software (SAS Institute Inc., Cary, NC, USA). Descriptive statistics were calculated for demographic variables and outcome measures. Categorical variables were compared using chi-square or Fisher’s exact tests, while continuous variables were analyzed using Student’s t-tests. Univariate logistic regression was used to evaluate associations between clinical variables and outcomes. Because only three VTE/DVT events occurred, the number of events was insufficient for meaningful multivariable modeling or adjusted comparison of prophylaxis groups. Accordingly, analyses of VTE/DVT should be considered exploratory, and failure to detect a statistically significant association should not be interpreted as evidence of equivalence between aspirin and no-aspirin groups. Statistical significance was defined as p-value <0.05.

## Results

From 2017 to 2023, a total of 1,069 shoulder arthroscopy patients and 203 shoulder arthroplasty patients met the inclusion criteria for this study. Significant differences were observed between the two cohorts in terms of age, sex, diabetes status, smoking history, and chronic anticoagulation use (Table 2). BMI and ASA prescribing rates were the only two categories that did not differ significantly. Overall, patients undergoing shoulder arthroplasty appeared to be less healthy than those undergoing arthroscopy.

**Table 2:** Demographic characteristics. **: Indicates statistical significance (p < 0.05). BMI = body mass index; ASA = acetylsalicylic acid

	Arthroscopy (n = 1,069)	Arthroplasty (n = 203)	P-value
Age (years, mean ± SD)	48.8 ± 15.4	66.9 ± 9.1	<0.0001**
Female/Male (%)	47.5/52.5	61.1/38.9	0.0004**
BMI (mean ± SD)	31.2 ± 7.4	32.0 ± 7.6	0.1413
Diabetes (%)	17.8	34.0	<0.0001**
On chronic anticoagulation (%)	19.0	43.4	<0.0001**
Smoking status			0.0014**
Never (%)	48.4	44.3	
Former (%)	27.1	38.9	
Current (%)	24.5	16.8	
ASA prescribed (%)	69.9	75.4	0.1191

Among all patients who underwent shoulder surgery (n = 1,272), postoperative ASA use was not associated with the occurrence of VTE/DVT (p = 0.9864). Our total number of VTE/DVTs reported was low, with only three (0.24%) patients experiencing this postoperatively (Table 3). Similarly, ASA use was not associated with unfavorable postoperative complications in patients undergoing shoulder surgery such as hematoma (p = 0.7254), reoperation (p = 0.2901), or readmission (p = 0.4326), with respective incidences of 0.55%, 1.57%, and 1.26%.

**Table 3:** Postoperative complications separated by postoperative ASA usage. Values are presented as n (%). P-values were calculated using Fisher’s exact test. Statistical significance was defined as p < 0.05. DVT = deep vein thrombosis; ASA = acetylsalicylic acid

Complication	Aspirin (n = 901)	No aspirin (n = 371)	P-value
DVT	2 (0.2%)	1 (0.3%)	0.986
Hematoma	4 (0.4%)	3 (0.8%)	0.725
Reoperation	11 (1.2%)	9 (2.4%)	0.290
Readmission	9 (1.0%)	7 (1.9%)	0.433

No significant difference was found whether ASA was used or not for rates of DVT, hematoma, reoperation, or readmission in either the arthroscopy (Figure 1) or arthroplasty (Figure 2) group except for reoperation (p = 0.021) in the arthroscopy group. Although statistically significant, the low absolute event rate limits clinical interpretation.

**Figure 1:**
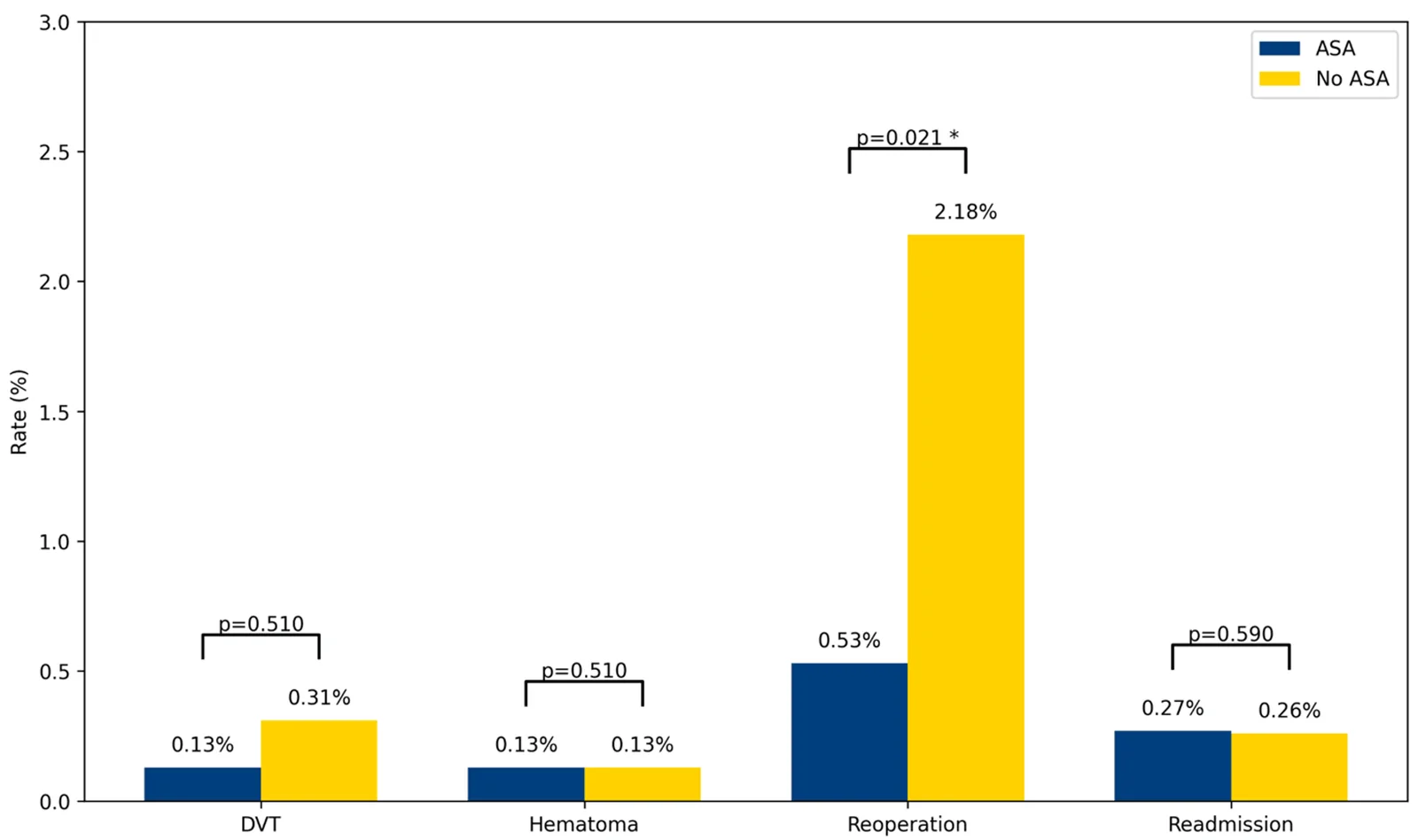
Arthroscopy event rates. *: Designates statistically significant with p < 0.05. DVT = deep vein thrombosis; ASA = acetylsalicylic acid

**Figure 2:**
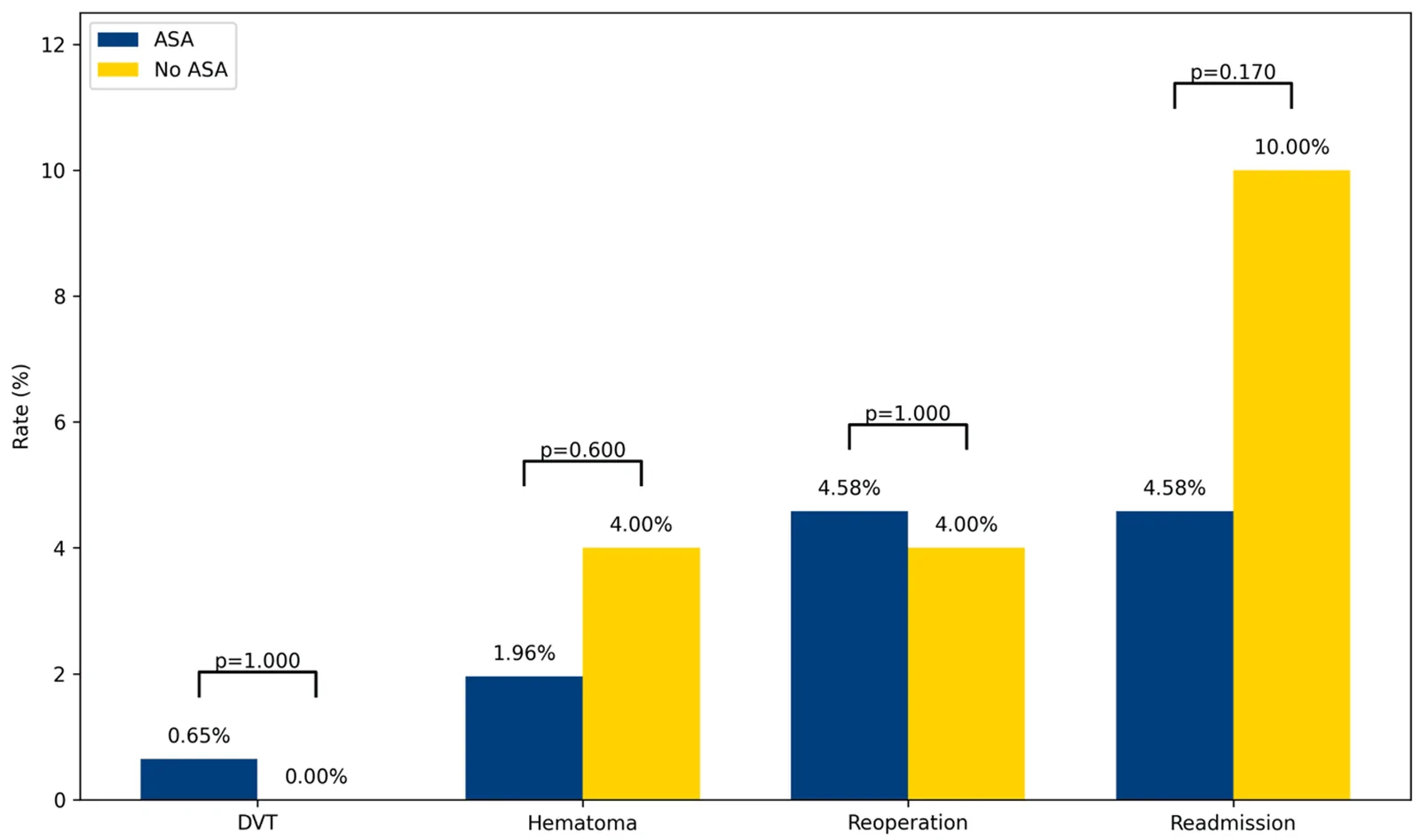
Arthroplasty event rates. *: Designates statistically significant with p < 0.05. DVT = deep vein thrombosis; ASA = acetylsalicylic acid

Univariate analysis was performed to evaluate whether age, sex, BMI, diabetes, tobacco use, or chronic anticoagulation use was associated with an increased risk of VTE/DVT, hematoma, reoperation, or readmission (Tables 4, 5). Among the variables examined, none were significantly associated with VTE/DVT. However, there were several variables that were independently associated with our other outcomes of interest. Chronic anticoagulation use was associated with increased readmission (odds ratio (OR) = 4.44; 95% confidence interval (CI) = 1.64-12.02; p = 0.0014) and reoperation (OR = 2.81; 95% CI = 1.15-6.85; p = 0.0176). Whereas older age (defined as greater than 65 years old) was significantly associated with postoperative hematoma (OR = 8.905; 95% CI = 1.72- 46.08) and readmission (OR = 6.02; 95% CI = 2.17- 16.72). These estimates represent unadjusted associations because multivariate analysis was not feasible given the low number of outcome events.

**Table 4:** Univariate analysis of patient characteristics by DVT and hematoma status. Values are presented as mean ± SD or n (%). **: Indicate statistical significance (p < 0.05). BMI = body mass index; DVT = deep vein thrombosis

Variable	No DVT	DVT	P-value	No Hematoma	Hematoma	P-value
Age, years	51.7 ± 16.0	56.0 ± 7.2	0.408	51.6 ± 16.0	66.4 ± 8.1	0.003**
Sex, n (%)	-	-	0.85	-	-	0.933
Female	631 (49.7)	1 (33.3)		629 (49.7)	3 (42.9)	
Male	638 (50.3)	2 (66.7)		636 (50.3)	4 (57.1)	
BMI, kg/m²	31.3 ± 7.4	30.7 ± 4.2	0.814	31.3 ± 7.4	30.9 ± 5.8	0.842
Diabetes, n (%)	-	-	0.678	-	-	0.337
Yes	260 (20.5)	0 (0.0)		257 (20.3)	3 (42.9)	
No	1009 (79.5)	3 (100.0)		1008 (79.7)	4 (57.1)	
Tobacco use, n (%)	-	-	0.623	-	-	0.31
Yes	664 (52.3)	1 (33.3)		660 (52.2)	5 (71.4)	
No	605 (47.7)	2 (66.7)		605 (47.8)	2 (28.6)	
Chronic anticoagulation, n (%)	-	-	0.071**	-	-	0.207
Yes	289 (22.8)	2 (66.7)		288 (22.8)	3 (42.9)	
No	980 (77.2)	1 (33.3)		977 (77.2)	4 (57.1)	

**Table 5:** Univariate analysis of patient characteristics with readmission and reoperation. Values are presented as mean ± SD or n (%). **: Indicates statistical significance (p < 0.05). BMI = body mass index

Variable	No readmission	Readmission	P-value	No reoperation	Reoperation	P-value
Age, years	51.5 ± 15.9	63.8 ± 15.9	0.008**	51.6 ± 16.0	54.3 ± 16.2	0.474
Sex, n (%)	-	-	0.885	-	-	0.906
Female	625 (49.8)	7 (43.8)		623 (49.8)	9 (45.0)	
Male	631 (50.2)	9 (56.3)		629 (50.2)	11 (55.0)	
BMI, kg/m²	31.3 ± 7.4	30.5 ± 4.7	0.504	31.3 ± 7.4	30.7 ± 5.7	0.608
Diabetes, n (%)	-	-	0.556	-	-	0.091
Yes	255 (20.3)	5 (31.3)		252 (20.1)	8 (40.0)	
No	1001 (79.7)	11 (68.8)		1000 (79.9)	12 (60.0)	
Tobacco use, n (%)	-	-	0.197	-	-	0.509
Yes	657 (52.3)	8 (50.0)		657 (52.5)	8 (40.0)	
No	599 (47.7)	8 (50.0)		595 (47.5)	12 (60.0)	
Chronic anticoagulation, n (%)	-	-	0.001**	-	-	0.018**
Yes	282 (22.5)	9 (56.3)		282 (22.5)	9 (45.0)	
No	974 (77.5)	7 (43.8)		970 (77.5)	11 (55.0)	

Out of the 1,069 patients who underwent arthroscopy, 748 (70.0%) received aspirin postoperatively, and out of 203 patients who underwent shoulder arthroplasty, 153 (75.4%) received aspirin postoperatively. There was no significant difference in DVT incidence between the arthroscopy and arthroplasty groups. However, patients who underwent arthroplasty experienced more postoperative complications, including significantly more hematomas (p < 0.0001), readmissions (p < 0.0001), and reoperations (p = 0.0004) compared with arthroscopy procedures (Table 6).

**Table 6:** Postoperative outcomes by surgery type and ASA use. **: Indicate statistical significance (p < 0.05). DVT = deep vein thrombosis; ASA = acetylsalicylic acid

Outcome	Arthroscopy ASA+ (n = 748)	Arthroscopy ASA- (n = 321)	Arthroscopy total (n = 1,060)	Arthroplasty ASA+ (n = 153)	Arthroplasty ASA- (n = 50)	Arthroplasty total (n = 203)	P-value
DVT	1 (0.13%)	1 (0.31%)	2 (0.19%)	1 (0.65%)	0 (0.00%)	1 (0.49%)	0.4107
Hematoma	1 (0.13%)	1 (0.13%)	2 (0.19%)	3 (1.96%)	2 (4.00%)	5 (2.46%)	<0.0001**
Reoperation	4 (0.53%)	7 (2.18%)	11 (1.03%)	7 (4.58%)	2 (4.00%)	9 (4.43%)	0.0004**
Readmission	2 (0.27%)	2 (0.26%)	4 (0.37%)	7 (4.58%)	5 (10.00%)	12 (5.91%)	<0.0001**

## Discussion

Shoulder arthroscopy and aspirin

The incidence of VTE after shoulder arthroscopy in our cohort was 0.19%, consistent with prior reports demonstrating rates generally below 0.25% [10-12,14]. Importantly, ASA use did not significantly influence VTE/DVT occurrence, hematoma formation, readmission, or reoperation. These results align with prior retrospective studies and systematic reviews suggesting that pharmacologic prophylaxis does not meaningfully alter thromboembolic risk in arthroscopic shoulder procedures [10-13].

Given the extremely low baseline event rate, our study did not demonstrate a statistically significant reduction in symptomatic VTE/DVT associated with postoperative aspirin following shoulder arthroscopy. However, the limited number of VTE/DVT events substantially limits statistical power and precludes definitive conclusions regarding comparative efficacy. Therefore, these findings should not be interpreted as demonstrating that aspirin prophylaxis is ineffective or unnecessary.

Shoulder arthroplasty and aspirin

In contrast to arthroscopy, patients undergoing shoulder arthroplasty experienced higher rates of postoperative complications, particularly hematoma, readmission, and reoperation. Despite this, the incidence of VTE/DVT remained low (0.49%), consistent with large database and systematic review data reporting rates between 0.24% and 2.6% [1,2,14]. Low-dose ASA after primary shoulder arthroplasty has been associated with low rates of symptomatic VTE/DVT and minimal complications [7]. Aspirin was not associated with increased bleeding-related events, and we did not detect a statistically significant association between aspirin use and symptomatic VTE/DVT. However, the small number of thromboembolic events limits our ability to evaluate comparative efficacy, and these findings should not be interpreted as demonstrating an absence of prophylactic benefit. Importantly, patient-specific factors were more predictive of adverse outcomes than aspirin use. Chronic anticoagulation independently increased the risk of readmission and reoperation, and age greater than 65 years was associated with increased hematoma and readmission rates. These associations emphasize that complication risk in shoulder arthroplasty is more closely related to comorbidity burden than to aspirin administration itself.

Shoulder arthroscopy and arthroplasty side by side

When directly compared, our cohorts highlight important contrasts. While both procedures demonstrated very low rates of postoperative VTE/DVT, the arthroplasty group experienced significantly higher rates of hematoma (2.46% vs. 0.19%), reoperation (4.43% vs. 1.03%), and readmission (5.91% vs. 0.37%). These findings parallel prior registry data and database studies showing that patients undergoing shoulder arthroplasty are typically older, have more comorbidities, and consequently sustain greater postoperative risks [1,4,14]. A statistically significant association between aspirin administration and thromboembolic events was not detected in either group. Given the limited event number, these findings cannot establish equivalence between prophylaxis strategies. Rather, they underscore the low absolute incidence of symptomatic VTE/DVT and support further investigation of risk-stratified prophylaxis strategies. In shoulder arthroplasty, prophylaxis decisions should be individualized based on patient-specific factors such as age, chronic anticoagulation status, and overall comorbidity profile.

Safety considerations

Aspirin was not associated with increased hematoma, readmission, or reoperation in our cohort. This is consistent with prior work demonstrating a favorable safety profile of low-dose aspirin compared with more potent anticoagulants [6,7]. Therapeutic anticoagulation has been associated with increased wound complications and infection following shoulder arthroplasty, reinforcing the importance of balancing thromboembolic prevention with bleeding risk [6]. Importantly, our results highlight that patient-specific variables, particularly chronic anticoagulation and advanced age, were more predictive of adverse outcomes than aspirin itself. These findings suggest that while aspirin appears safe, it should not be considered universally benign; rather, its prophylactic role should be weighed against patient comorbidity and surgical context.

Limitations

This study has several important limitations. First, its retrospective, single-institution design introduces the potential for selection bias, incomplete documentation, and residual confounding. Postoperative aspirin administration was not randomized and was based on surgeon practice and clinical judgment, creating the potential for confounding by indication. Patients selected to receive aspirin may therefore have differed systematically from those who did not receive aspirin in measured or unmeasured VTE risk factors.

Most importantly, only three symptomatic VTE/DVT events occurred in the entire cohort. Although this finding reinforces the low absolute incidence of symptomatic thromboembolic events following shoulder surgery, the small number of events substantially limits statistical power and precluded meaningful multivariable adjustment for the primary outcome. This is a recognized challenge across shoulder literature, as most investigations report similarly low event rates [10-12,14].

Accordingly, the absence of a statistically significant association between aspirin use and VTE/DVT should not be interpreted as evidence of equivalence or definitive evidence that aspirin provides no prophylactic benefit. A clinically meaningful difference may have gone undetected because of a Type II error.

Although four surgeons contributed patients, surgeon-level practice patterns varied, including aspirin selection, dosing (81 mg twice daily or 325 mg daily), and duration (two to four weeks). The study was not designed or powered to compare individual aspirin regimens. Additionally, the arthroscopy and arthroplasty cohorts included heterogeneous procedures with potentially different operative times, postoperative immobilization requirements, and baseline thromboembolic risks. Mechanical prophylaxis and postoperative mobilization were not reliably captured in the retrospective record and could not be incorporated into the analysis. Other unmeasured VTE risk factors may similarly have influenced treatment selection and outcomes.

Finally, we did not directly compare aspirin with alternative pharmacologic agents such as LMWH or direct oral anticoagulants. Follow-up was limited to 90 days, which was intended to capture clinically relevant postoperative events but may not identify later occurrences. Larger prospective multicenter studies with standardized prophylaxis protocols and sufficient event numbers for adjusted analyses are necessary to determine whether aspirin provides a clinically meaningful prophylactic benefit following shoulder surgery and to identify which patients are most likely to benefit.

## Conclusions

Symptomatic VTE/DVT was uncommon following shoulder surgery in this cohort, with low observed rates after both arthroscopy and arthroplasty. Postoperative aspirin use was not significantly associated with a reduction in VTE/DVT or with increased hematoma, readmission, or reoperation. However, the small number of VTE/DVT events and nonrandomized selection of aspirin prophylaxis substantially limit conclusions regarding comparative efficacy, and the absence of a statistically significant association should not be interpreted as evidence of equivalence or absence of prophylactic benefit. These findings underscore the low absolute incidence of symptomatic VTE/DVT following shoulder surgery and support individualized assessment of thromboembolic and bleeding risk when considering postoperative chemoprophylaxis. Larger prospective studies with standardized prophylaxis protocols are needed to determine which patients, if any, derive meaningful benefit from routine aspirin prophylaxis.
